# The surgical treatment of idiopathic abnormal uterine bleeding: An analysis of 88 000 patients from the French exhaustive national hospital discharge database from 2009 to 2015

**DOI:** 10.1371/journal.pone.0217579

**Published:** 2019-06-11

**Authors:** Lucie de Léotoing, Gwendoline Chaize, Jérôme Fernandes, Dusan Toth, Philippe Descamps, Gil Dubernard, Thomas Lafon, Ludovic Lamarsalle, Hervé Fernandez

**Affiliations:** 1 HEVA, Lyon, France; 2 OC Santé, Montpellier, France; 3 Clinique Saint Germain, Brive La Gaillarde, France; 4 C.H.U. Angers, France; 5 Hôpital de la Croix-Rousse, Lyon, France; 6 Hôpital Bicêtre, Le Kremlin-Bicêtre, France; Universita degli Studi dell'Insubria, ITALY

## Abstract

**Objective:**

The objective of the study was to compare success rates, complications and management costs of different surgical techniques for abnormal uterine bleeding (AUB).

**Methods:**

This was a retrospective analysis of the French national hospital discharge database. All hospital stays with a diagnostic code for AUB and an appropriate surgical procedure code between 2009 and 2015 inclusive were identified, concerning 109,884 women overall. Outcomes were compared between second generation procedures (2G surgery), first-generation procedures (1G surgery), curettage and hysterectomy. Clinical outcomes were treatment failure and complications during the follow-up period. Costs were attributed using standard French hospital tariffs.

**Results:**

7,863 women underwent a 2G procedure (7.2%), 39,935 a 1G procedure, (36.3%), 38,923 curettage (35.4%) and 23,163 hysterectomy (21.1%). Failure rates at 18 months were 9.9% for 2G surgery, 12.7% for 1G surgery, 20.6% for curettage and 2.8% for hysterectomy. Complication rates at 18 months were 1.9% for 2G surgery, 1.5% for 1G surgery, 1.4% for curettage and 5.3% for hysterectomy. Median 18-month costs were € 1 173 for 2G surgery, € 1 059 for 1G surgery, € 782 for curettage and € 3 090 for hysterectomy.

**Conclusion:**

Curettage has the highest failure rate. Hysterectomy has the lowest failure rate but the highest complication rate and is also the most expensive. Despite good clinical outcomes and relatively low cost, 1G and 2G procedures are not widely used. Current guidelines for treatment of AUB are not respected, the recommended 2G procedures being only used in <10% of cases.

## Introduction

Abnormal uterine bleeding (AUB) is defined as at least one of the following three conditions (1) menstrual periods lasting more than seven days, (2) periods involving blood loss greater than 80 ml or (3) a PBAC (pictorial menstrual blood loss assessment chart) score ≥ 100.[1] It is thought that between twenty and thirty percent of women of reproductive age present with AUB at some time.[1, 2] A recent survey in the general population of five European countries reported that 27.2% had experienced two or more symptoms of AUB within the previous year.[2] The prevalence of AUB increases with age up until the menopause.[1] Although AUB may occur secondary to another underlying pathology, frequently leiomyoma, malignancies or coagulopathies, in many cases no underlying cause can be identified and it is considered idiopathic. [3, 4]

Health-related quality of life is significantly deteriorated in patients with AUB.[5, 6] In the European epidemiological survey,[2] over half the patients reporting AUB stated that their symptoms had a major or moderate negative impact on their sexual life, physical activities and productivity at work.[2] Using a general quality of life measure (Short-Form 36 Health Survey), scores for women with AUB were below the 25th percentile of national norms.[5] A relatively old (2001) study from Finland has reported that 20% of women with objective evidence of AUB had missed work because of AUB over the previous six months.[7]

Treatment of idiopathic AUB can offer effective relief of symptoms and improvement of quality of life.[5] The choice of treatment depends on the severity of the symptoms and whether it is desired to preserve fertility. Both medical and surgical treatments are available. The former include oral contraceptives (oestrogen plus progestin or only progestin), tranexamic acid, mefenamic acid, gonadotropin-releasing hormone analogues, and intrauterine systems (IUS) containing lævonorgestrel. Surgical procedures include curettage and hysterectomy, as well as more targeted hysteroscopic procedures such as resection, roller-ball, and laser ablation (first generation surgical procedures).[8] More recently, a second generation of surgical procedures has been developed, including thermal balloon, cryoablation, and microwave or radiofrequency ablation. Systematic reviews and meta-analyses suggest that second-generation endometrial ablation procedures seem to be as effective as first-generation procedures and present fewer complications.[9, 10] They could be more often performed under local anaesthesia or cervical block and requires shorter operating times.[9, 10] For these reasons, second generation surgical procedures have been considered more cost-effective, despite an observed higher rate of equipment failure and inability to complete the procedure.[9, 10]

According to current French practice guidelines,[1] as well as guidelines from other countries such as the Netherlands,[11] England and Wales,[12] the USA[13] and Canada,[14] first-line treatment of idiopathic AUB (as defined by COEIN classification) should be pharmacological. For women who do not wish to become pregnant in the future and who have idiopathic AUB, the long-term efficacy of conservative surgical treatment is greater than that of oral medical treatment,[1] and should be proposed if treatment with tranexamic acid or placement of a lævonorgestrel-containing IUS is not sufficiently effective.[1] The guidelines also state that surgical treatments of choice include second-generation endometrial ablation techniques, or, if necessary, first-generation techniques. A first-line hysterectomy is not recommended in this context. In the guidelines, curettage is only considered acceptable except when conservation of fertility is desired, and its efficacy is considered random and temporary.[1]

The objective of this study was to describe the evolution of four surgical procedures performed for the treatment of idiopathic AUB (second generation, curettage, first-generation and hysterectomy) and to compare their success rates, severe complications and management costs.

## Materials and methods

### Ethics

The study was conducted in accordance with relevant international and French regulatory requirements. Since this was a retrospective study of an anonymised database and had no influence on patient care, ethics committee approval was not required. Use of the PMSI-MCO database for this type of study has been approved by the French national data protection agency (CNIL; annual authorisation #1419102 v7–2015-111111-56-18 / order M14N056 and M14L056).

### Study design

This retrospective study analysed data extracted from the French Hospital medical information database (*Programme de Médicalisation des Systèmes d’Information* [PMSI]). The study population consisted of all patients hospitalised for a surgical treatment of idiopathic AUB in France from 2009 to 2015. Data extraction and analysis followed the guidelines for exploitation of the PMSI database published by the French Health Ministry [15].

### PMSI database

The French PMSI-MCO (*Médecine*, *Chirurgie*, *Obstétrique*) is an exhaustive medico-administrative hospital discharge database which covers all overnight or day hospitalisations in the public and private sectors involving short-term stays in medical, surgical or obstetric facilities.[16] The database is used by the French health authorities to manage funding of all hospital services. Each year, almost 30 million stays by over 13 million patients are reported in this database. Information is exhaustive for all public and private hospitals in France and no sampling is performed.

In the PMSI, all hospital stays are attributed to a diagnosis-related group (DRG) reflecting the reasons for hospitalisation. These are identified by the physician through International Classification of Diseases, 10th revision (ICD-10) codes,[17] either as principal diagnoses (PD: the condition for which the patient was hospitalised), related diagnoses (RD: any underlying condition which may have been related to the PD) or as significantly-associated diagnoses (SAD: comorbidities or complications which may affect the course or cost of hospitalisation). All procedures (such as surgery) performed during the stay are categorised and identified according to the French Classification of Medical Procedures (CCAM, *Classification Commune des Actes Médicaux*).

### Study population

All hospital stays in France with at least one CCAM code for AUB surgery associated with at least one ICD-10 code for idiopathic AUB documented as a PD or RD of the medical unit where the CCAM surgery was coded were identified from the PMSI-MCO database between 1^st^ January 2009 and 31^st^ December 2015. These codes are listed in S2 Table and S3 Table, and exclude post-menopausal bleeding. Only women aged between 35 and 55 years were retained. The reason for the lower age limit of 35 years was to exclude a majority of women who wished to remain fertile, since all the procedures evaluated (with the exception of curettage) are only indicated in women who no longer wish to have children. As this is not documented in the PMSU database, the age threshold was used as a surrogate criterion. All stays were classified according to the type of idiopathic AUB surgery that was performed into one of four classes: (i) first generation (1G) endometrial ablation or resection techniques, such as loop resection or roller ball, (ii) second generation (2G) endometrial ablation techniques such as radiofrequency ablation or balloon thermodestruction, (iii) hysterectomy, and (iv) curettage.

Patients already operated on for AUB surgery were excluded, in order to include incident patients only. A retrospective linkage until 2006 was performed to search for previous AUB surgery, defined as the presence of at least one of the CCAM codes for AUB surgery. An algorithm was developed with the scientific committee in order to exclude any patient identified as presenting comorbidities that would introduce bias in the results, such as breast or colorectal cancer at inclusion or before inclusion (until 2006), von Willebrand disease at inclusion, before inclusion (until 2006) or during follow-up (until end 2015), stays combining several idiopathic AUB surgeries, preventing assignment to one of the four groups, any concomitant pathology that could also cause bleeding (myoma, gynaecological cancers, alcoholic liver disease, gynaecological and pelvic infections and inflammation, endometriosis, uro-gynaecological prolapse, fistulas, polyps and dysplasia, infertility, pregnancy, spina bifida, blood diseases), artificial insemination attempts after curettage in 35–45 year old patients.

Patients operated on before mid-2014 were followed for at least 18 months from their surgery and up to 5 years (for patients included in 2009 and 2010). Outcome over the period from the intervention until 31^st^ December 2015 (or death, if the patient died before the end of the follow-up period) was dichotomised into success and failure. Success was defined as the absence of all three of the following failure criteria: (i) other surgical intervention (1G, 2G, curettage, hysterectomy) for AUB, (ii) persistent idiopathic AUB requiring medical management in hospital or (iii) pregnancy.

Severe complications were defined as medical events linked to idiopathic AUB surgery and requiring hospitalisation of the patient and were tracked until the 31^st^ December 2015, death or until failure. Certain complications were considered to be acute (within 30 days), namely inflammatory disorders, haemoperitoneum or systemic complications (anaemia, shock, any complication directly linked to a surgical intervention, pain, infection, blood supply). Other complications located within the uro-genito-abdo-pelvic sphere could occur at any time after surgery location, such as adhesions, foreign body, pain, ventral hernia, renal failure, intestinal obstruction, and other digestive disorders, urological/genital disorders. These complications were identified by ICD10 codes. Any surgical or interventional procedures occurring between AUB surgery and the complication were tracked, as they could potentially be the reason of complication. Only complications in the absence of any intercurrent procedure were considered as certainly related to AUB surgery and were retained for the analysis.

### Data collection

The socio-demographic characteristics of patients were collected at inclusion (age). For each initial index hospital stay, the distribution per type of hospitalisation was analysed: overnight or day hospitalisations. For patients operated on before mid-2014, outcome and complication rates was documented at 18, 24 and 60 months.

Total in-hospital medical resource consumption associated with all hospitalisations occurring within the period from the initial index hospitalisation up until any stay for failure or complication was documented. Medications prescribed or delivered during outpatient consultations was not considered, as they were not documented in the database.

### Costing

*Per capita* costs at 18 months after idiopathic AUB surgery were determined for each cohort. Costing was restricted to direct costs and determined from the perspective of the French social security system (National Health Insurance; NHI). Costs were attributed from official French national tariffs for medical acts applicable in France from 2009 to 2015, and were expressed in 2017 Euros by adjustment by the a relevant Consumer Price Index. A standard national tariff was applied to each hospitalisation based on the DRG code attributed in the PMSI database. These standard tariffs include medical and related procedures, nursing care, treatments (except specific expensive drugs and implants), food and accommodation, and investment costs for hospitalised patients. Additional costs per day of hospitalisation in an intensive care unit were added to the DRG tariffs when appropriate. For private hospitals, where physicians are reimbursed on a fee-for-service basis, physician fees were identified from the ENCC (*Echelle Nationale des Coûts à méthodologie Commune*, the French observatory of real-world spending on healthcare) and added to the DRG tariffs. For public hospitals, where physicians are salaried, physician fees are included in the DRG tariff, which takes it into account all personnel costs. Expensive drugs and implants were costed using the retail price listed in the public FICHCOMP database and the official tariff for the private database. For expensive drugs and implants used in 2009, their costs were attributed on the basis of the mean FICHCOMP-derived costs in public hospitals for 2009, as 2009 FICHCOMP was not available for private hospitals.

### Statistical analysis

Categorical data were expressed as proportions and analysed by using the χ2 test or the Fisher exact test. Univariate statistical tests for continuous data included tests of mean differences with Student’s *t*-test. Occurrence of outcomes (treatment failure or complications) were followed over time using Kaplan-Meier survival curves and outcome rates compared between 2G procedures (as the reference) and the three other procedures using Mantel-Cox log-rank test (unadjusted univariate analysis). A P values of < .05 was used to determine the statistical significance of all tests used. Analyses were performed by using the R statistical software (version 3.2.3).

## Results

### Study population

**Fig 1:**
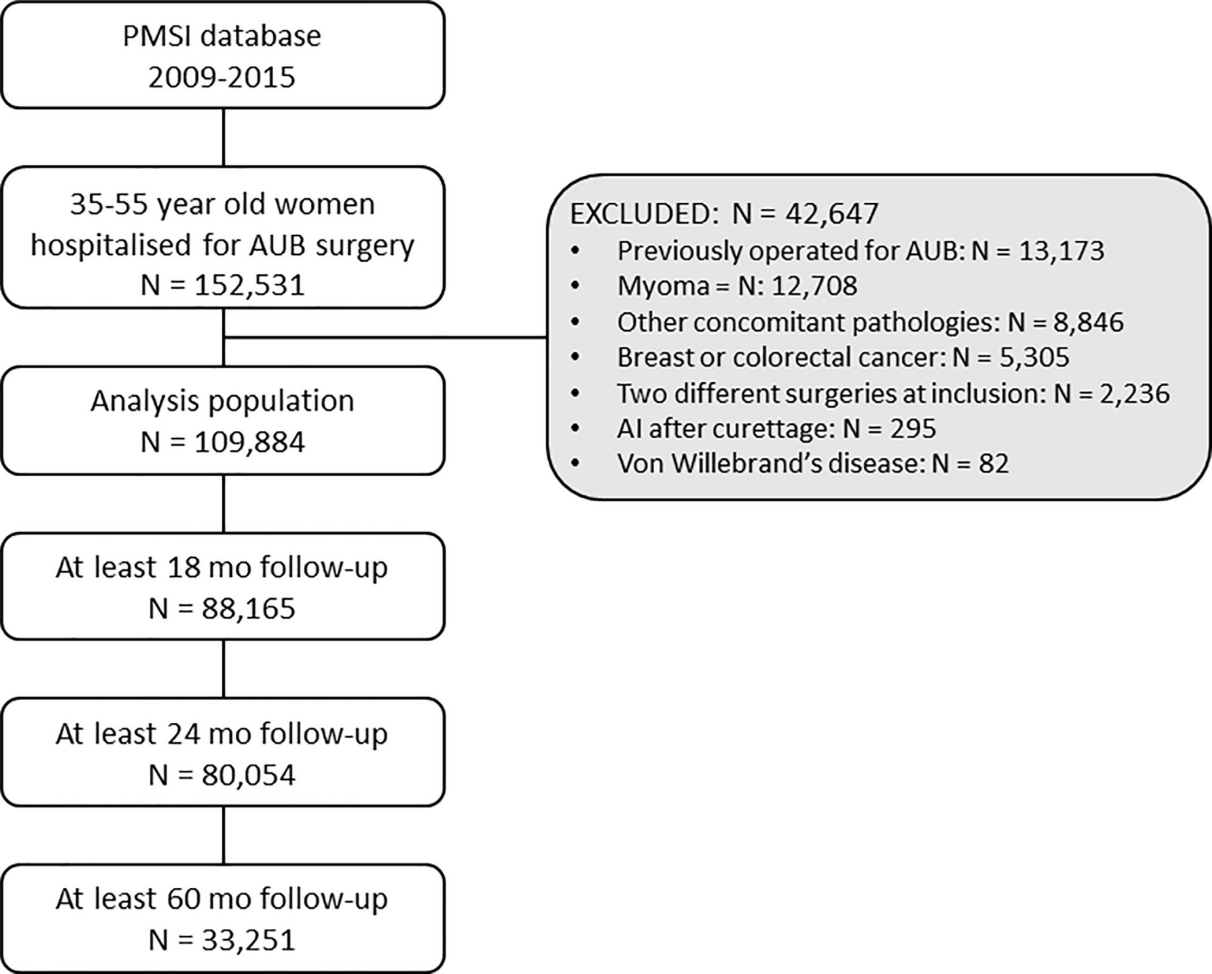
Flow diagram for the selection of the study population. AUB: idiopathic abnormal uterine bleeding; AI: artificial insemination.

Between 1^st^ January 2009 and 31^st^ December 2015, 152,531 35–55 year old women hospitalised for surgery for idiopathic AUB were identified in the PMSI database, of whom 42,647 presented at least one exclusion criterion and were excluded. The cohort of patients available for analysis thus consisted of 109,884 patients. Of these, 88,165 could be followed for 18 months, 80,054 for 24 months and 33,251 for 60 months. The flow diagram for the selection of patients is illustrated in Fig 1.

The mean age of the participants was 46 years, with no difference in age between surgery types (S1 Table).

### Surgery for idiopathic abnormal uterine bleeding

**Fig 2:**
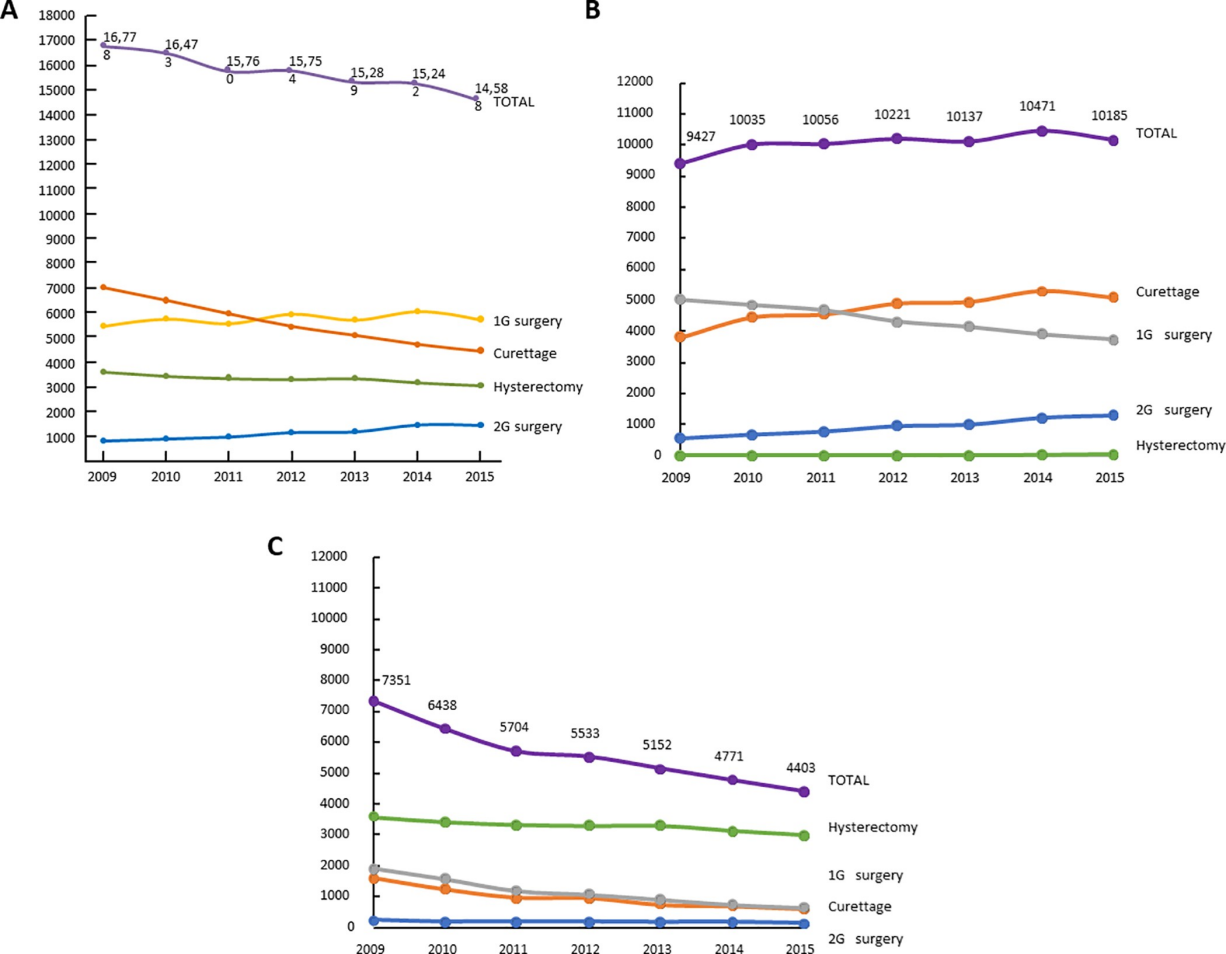
Evolution of surgery for idiopathic abnormal uterine bleeding between 2009 and 2015. A: All procedure. B: Day hospitalisation procedures. C: Full hospitalisation procedures.

In the analysis population, 7,863 women underwent a 2G surgical procedure (7.2%), 39,935 a 1G procedure (36.3%), 38,923 curettage (35.4%) and 23,163 hysterectomy (21.1%). The number of women undergoing surgery decreased by 13% between 2009 and 2015 (Fig 2). This evolution was due to a decrease in the number of women undergoing curettages (-37%) and hysterectomies (-15%) with a concomitant increase in 2G surgical procedures (+80%). The number of 1G procedures remained relatively stable (+5%). Almost all hysterectomies were performed during an overnight stay, whereas most of the other procedures (around 80%) were performed in day hospitalisation (S1 Table). The proportion of all procedures performed in day hospitalisation increased somewhat from 2009 to 2015.

### Treatment outcome

The rate of treatment failure at 18 months ranged from 2.8% in women who had undergone hysterectomy to 20.6% in patients having undergone curettage (Table 1). The risk of failure at 18 months after surgery was significantly higher (p<0.0001) in women undergoing 1G surgical procedures (HR: 1.30) and curettage (HR: 2.21) compared with women undergoing 2G procedures. A similarly elevated risk (p<0.0001) was also observed at 24 months for both techniques (HR: 1.26 for 1G procedures and 2.21 for curettage), and at 60 months for curettage only (HR 1.75; p<0.0001).

**Table 1:** Treatment failures and complications in women undergoing surgery for idiopathic abnormal uterine bleeding.

	2G Surgery	1G Surgery	Curettage	Hysterectomy
18 months	N = 5 731	N = 31 476	N = 32 307	N = 18 651
Failures	569 (9.9%)	4 009 (12.7%)*	6 660 (20.6%)*	519 (2.8%)*
Complications	108 (1.9%)	480 (1.5%)	452 (1.4%)	995 (5.3%)*
24 months	N = 5 009	N = 28 240	N = 29 833	N = 16 972
Failures	585 (11.7%)	4 118 (14.6%)*	6 885 (23.1%)*	485 (2.9%)*
Complications	110 (2.2%)	509 (1.8%)	469 (1.6%)	981 (5.8%)*
60 months	N = 1 682	N = 11 130	N = 13 417	N = 7 022
Failures	318 (18.9%)	2 390 (21.5%)	4 074 (30.4%)*	198 (2.8%)*
Complications	77 (4.6%)	304 (2.7%)*	353 (2.6%)*	555 (7.9%)*

*Statistically significant difference vs 2G surgery (p<0.0001; Cox model)

### Severe complications

The rate of severe complications at 18 months ranged from 1.4% in women who had undergone curettage to 5.3% in patients having undergone hysterectomy (Table 1). Women undergoing hysterectomy were at significantly higher risk for severe complications at 18 months after surgery than women undergoing 2G procedures (HR 2.89; p<0.0001). A similarly elevated risk (p<0.0001) was observed at 24 (HR: 2.69) and 60 months (HR: 1.77). The principal complications observed in women undergoing hysterectomy were pain in the urogenital or abdominopelvic areas, infections and surgical complications.

### Cost of hospitalisation

**Fig 3:**
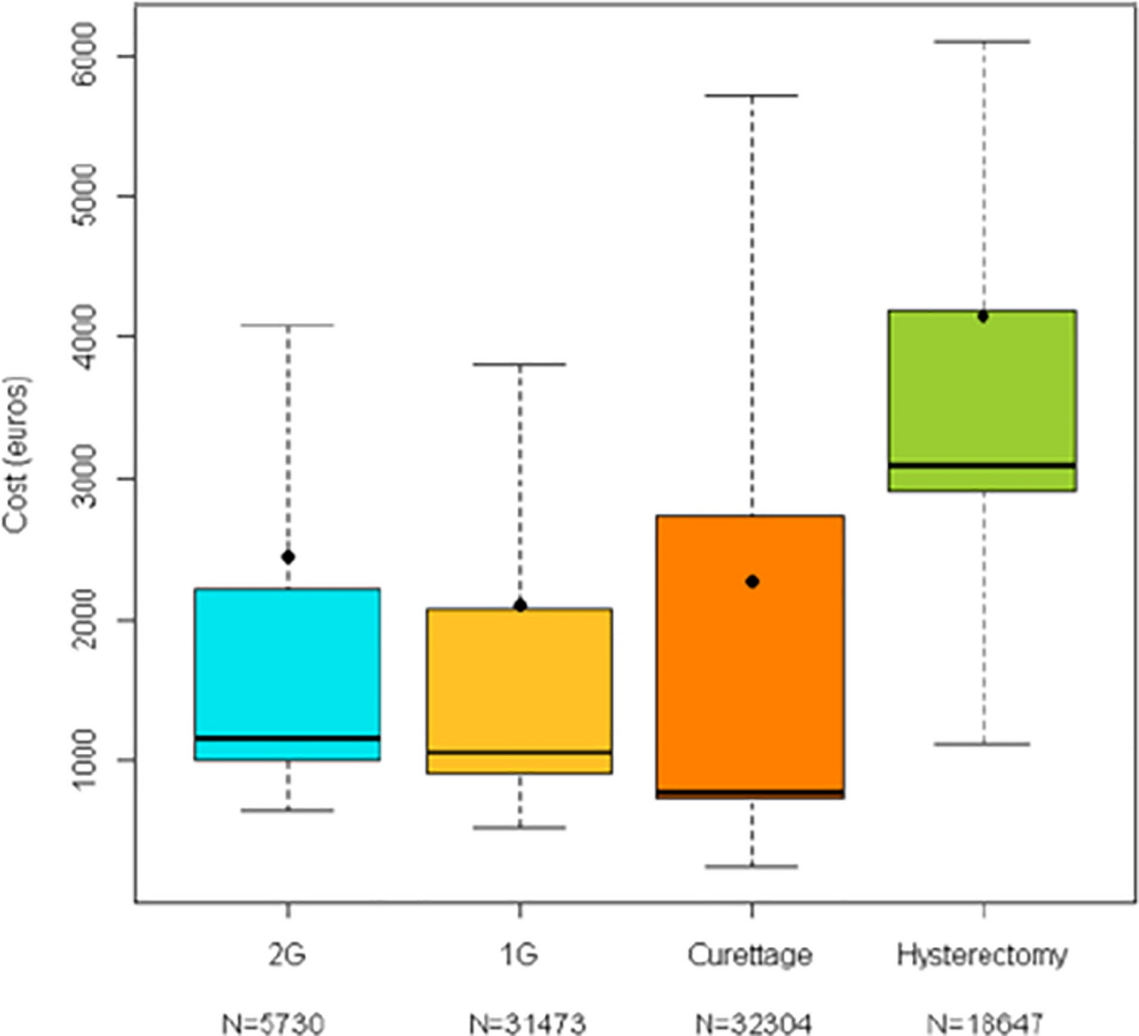
Box and whisker plots of total per capita 18-month costs of surgery for idiopathic abnormal uterine bleeding. The horizontal line represents the median cost and the round symbol the mean value. The coloured box indicates the interquartile range, and the upper and lower lines the minimum and maximum values.

Mean and median total *per capita* hospitalisation costs are presented in Table 2 and displayed as box and whisker plots in Fig 3. Mean and median costs were highest for women having undergone hysterectomy, almost two-fold higher than for the other types of procedure at all time-points.

**Table 2:** *Per capita* total 18-month costs of management of women undergoing surgery for idiopathic abnormal uterine bleeding.

	2G Surgery	1G Surgery	Curettage	Hysterectomy
18 months	N = 5 730	N = 31 473	N = 32 304	N = 18 647
Mean	€ 2 448 ± 8 254	€ 2 100 ± 3 476*	€ 2 275 ± 5 278*	€ 4 157 ± 3 535*
Median	€ 1 173 [638–489 846]	€ 1 059 [529–157 965]	€ 782 [244–424 261]	€ 3 090 [1 129–112 002]
IQR	€ 1 002–2 231	€ 913–2 075	€ 741 - € 2 732	€ 2 909 - € 4 189

Mean values are presented with their standard deviations and median values with the range. IQR: interquartile range.

*Statistically significant difference vs 2G Surgery (p<0.0001; *t*-test)

## Discussion

In this study, 109,884 women between 35 and 55 years old undergoing surgery for uncomplicated idiopathic AUB between 2009 and 2015 were identified, which is the largest cohort of surgically treated idiopathic AUB patients ever reported. With respect to the type of surgical procedure used, current practice does not follow the French guidelines [1]. Curettage and hysterectomy, neither of which is recommended as first-line treatment options, account for over half the surgical procedures performed, in spite of the fact that hysterectomy is over twice as expensive as other procedures and curettage has a two-fold higher failure rate. Second-generation procedures are the least frequently used, accounting for nor more than ten percent of all procedures. The number of women undergoing surgery for idiopathic AUB decreased by 13% between 2009 and 2015. This was essentially driven by a decrease in the number of women undergoing curettage, which decreased by 37%. A concomitant doubling in the number of 2G procedures over the same period was insufficient to compensate for the fall in the number of curettages. In addition, sales of IUD in France decreased over the same period [18], which means that a shift to non-surgical interventions cannot account for the fall in the number of women undergoing surgery for idiopathic AUB.

The principal strengths and weaknesses of the study derive from the use of the PMSI database as the data source. Among the strengths, the database is exhaustive and all cases of surgery for idiopathic AUB will have been captured, and their hospital follow-up tracked comprehensively. Moreover, the large number of cases documented allows the study outcomes to be determined with precision. Bias due to sampling errors and to loss to follow-ups should be minimal (due to the comprehensiveness of the database) and coding errors are believed to be rare. Nonetheless, since the tariffs for 1G and 2G procedures are different, it is possible that certain 2G procedures will have been coded as 1G procedures in order to benefit from more advantageous reimbursement. A major weakness is that the reasons for the choice of specific surgical acts are not documented, and this is a particular issue in imputing causal links between surgery and complications. However, the objective of the study was not to analyse the reasons underlying the choice of surgical procedure, but to describe the surgeries actually performed for the treatment of idiopathic AUB. Moreover, the database does not include data on histological findings on the surgery material, so the diagnosis of idiopathic AUB could not be ascertained in this way. In addition, the PMSI database is only available since 2006, and for this reason, women who had undergone surgery prior to this date may have erroneously been considered as incident cases. Finally, the PMSI database only contains information on care delivered in hospitals, and thus care provided in the community is not identified, which might underestimate the real cost and precludes obtaining an exhaustive treatment history for each patient.

It is important to note that this study was restricted to women with idiopathic AUB aged 35 – 55 years old. The lower age threshold was set in order to discourage inclusion of women wishing to remain fertile, as the procedures evaluated in the study (except curettage) are reserved for women who no longer wish to have children. Since the age criterion is not a perfect one, it is quite possible that certain women over 35 who still wanted to have children were included, which would have inflated the number of women undergoing curettage. Similarly, younger women who did not wish to have children and may have undergone one of the three other procedures will not have been captured. It is quite possible that the pattern of surgical procedures used may be different in younger women who can expect a longer future childbearing potential or in women with AUB due to other causes such as leiomyoma. In this context, the causes of AUB are likely to be different in younger women compared to the population of women relatively advanced in their reproductive life evaluated here.[19, 20]

Practice guidelines in France,[1] as well as in England and Wales [12] and in the Netherlands [11] all recommend 2G procedures as the preferred surgical option for women with idiopathic AUB who do not respond to pharmacological treatment. Notably, the guidelines from the Netherlands specifically recommend the use of radiofrequency ablation.[11] These recommendations reflect the findings of a number of meta-analyses and systematic reviews that have demonstrated the advantages of such procedures over hysterectomy or 1G procedures.[9, 10, 21] The outcomes observed in the present study suggest that 2G procedures are associated with lower severe complication rates and lower costs than hysterectomy, and higher success rates than curettage or 1G procedures. Although severe complication rates for hysterectomy may appear high, they are consistent with recent published data [22–24]. The situation in France lags behind that in certain other countries. For example, 2G procedures already accounted for around one-third of all surgery for idiopathic AUB in England in 2005.[25] One possible reason for the low rate of use in France is that these procedures are less well reimbursed, and there is thus a financial incentive for hospitals to encourage 1G procedures. Although this study was not a formal cost-effectiveness analysis, it appears that 1G and 2G procedures seem to be the most cost-effective strategies. Curettage, 1G, and 2G procedures all have similar costs. Curettage is the least effective strategy with the highest rate of failure, whereas, hysterectomy has the lowest rate of failure but the highest rate of severe complications and is also the most expensive strategy. The 3% failure rate in patients with hysterectomy seems somewhat surprising as the part of sub-total hysterectomies cannot explain it (7% of these 3%) and they could be due to coding errors. The failure rate for ablative procedures has been reported to increase with the length of time since the intervention [26]. Although it would have been possible to stratify patients by age group in order to determine post-procedure failure as a function of time, this was not performed due to the limited age range of the patients evaluated. Given this age distribution, any bias related to this would be expected to be minimal.

The widespread use of curettage is surprising since, according to the 2010 French guidelines,[1] it is only considered acceptable when preservation of fertility is required. No information is available in the PMSI database on whether the women treated desired to remain fertile or not, but given the age group included (35–55 years), it may be considered that participants would be less likely to wish to remain fertile than younger women. In the 2013 Netherlands guidelines [11] and the 2018 Anglo-Welsh guidelines,[12] curettage is no longer recommended in any context. Further research is needed to identify why curettage remains so widely used in France and to identify barriers to its replacement by more effective surgical procedures.

Switching to 2G procedures would not require specific training for surgeons, and in the case of curettage, would provide better outcomes for patients at little incremental cost and, in the case of hysterectomy, would lead to considerable cost savings. Increasing the DRG tariffs for 2G procedures could foster this switch.

In conclusion, this study shows that practice guidelines for treatment of idiopathic AUB are not respected, the recommended 2G procedures being only used in ten percent of cases. Further research should concentrate on comparing the clinical and economic consequences of current practice for idiopathic AUB surgery in observational clinical studies, not only in France but also in other countries where current practice guidelines may not be respected.

## Supporting information

S1 Table Age and hospitalisation in the study population. (DOCX)

S2 Table CCAM codes for AUB surgery. (DOCX)

S3 Table ICD-10 codes for idiopathic AUB. (DOCX)

## References

[1] Marret H, Fauconnier A, Chabbert-Buffet N, Cravello L, Golfier F, Gondry J, et al (2010) Clinical practice guidelines on menorrhagia: management of abnormal uterine bleeding before menopause. Eur J Obstet Gynecol Reprod Biol 152:133–7. Epub 2010/08/07. 10.1016/j.ejogrb.2010.07.016 . 20688424

[2] Fraser IS, Mansour D, Breymann C, Hoffman C, Mezzacasa A, Petraglia F. (2015) Prevalence of heavy menstrual bleeding and experiences of affected women in a European patient survey. Int J Gynaecol Obstet 128:196–200. Epub 2015/01/30. 10.1016/j.ijgo.2014.09.027 . 25627706

[3] Bacon JL. (2017) Abnormal Uterine Bleeding: Current Classification and Clinical Management. Obstet Gynecol Clin North Am 44:179–93. Epub 2017/05/14. 10.1016/j.ogc.2017.02.012 . 28499529

[4] Lagana AS, Vergara D, Favilli A, La Rosa VL, Tinelli A, Gerli S, et al (2017) Epigenetic and genetic landscape of uterine leiomyomas: a current view over a common gynecological disease. Arch Gynecol Obstet 296:855–67. Epub 2017/09/07. 10.1007/s00404-017-4515-5 . 28875276

[5] Liu Z, Doan QV, Blumenthal P, Dubois RW. (2007) A systematic review evaluating health-related quality of life, work impairment, and health-care costs and utilization in abnormal uterine bleeding. Value Health 10:183–94. Epub 2007/05/30. 10.1111/j.1524-4733.2007.00168.x . 17532811

[6] Karlsson TS, Marions LB, Edlund MG. (2014) Heavy menstrual bleeding significantly affects quality of life. Acta Obstet Gynecol Scand 93:52–7. Epub 2013/11/26. 10.1111/aogs.12292 . 24266506

[7] Hurskainen R, Aalto AM, Teperi J, Grenman S, Kivela A, Kujansuu E, et al (2001) Psychosocial and other characteristics of women complaining of menorrhagia, with and without actual increased menstrual blood loss. Bjog 108:281–5. Epub 2001/04/03. . 11281469 10.1111/j.1471-0528.2001.00040.x

[8] Vitale SG, Sapia F, Rapisarda AMC, Valenti G, Santangelo F, Rossetti D, et al (2017) Hysteroscopic Morcellation of Submucous Myomas: A Systematic Review. Biomed Res Int 2017:6848250 Epub 2017/09/28. 10.1155/2017/6848250 . 28948169 PMC5602656

[9] Kroft J, Liu G. (2013) First- versus second-generation endometrial ablation devices for treatment of menorrhagia: a systematic review, meta-analysis and appraisal of economic evaluations. J Obstet Gynaecol Can 35:1010–9. Epub 2013/11/20. 10.1016/S1701-2163(15)30789-1 . 24246401

[10] Lethaby A, Hickey M, Garry R, Penninx J. (2009) Endometrial resection / ablation techniques for heavy menstrual bleeding. Cochrane Database Syst Rev:Cd001501. Epub 2009/10/13. 10.1002/14651858.CD001501.pub3 . 19821278

[11] Nederlandse Vereniging voor Radiologie, Nederlandse Vereniging voor Obstetrie en Gynaecologie. Hevig menstrueel bloedverlies (HMB). 2013.

[12] National Institute for Health and Care Excellence Heavy menstrual bleeding: assessment and management. London: NICE, 2018. 29634173

[13] Wheeler TL 2nd, Murphy M, Rogers RG, Gala R, Washington B, Bradley L, et al (2012) Clinical practice guideline for abnormal uterine bleeding: hysterectomy versus alternative therapy. J Minim Invasive Gynecol 19:81–8. Epub 2011/11/15. 10.1016/j.jmig.2011.10.001 . 22078016

[14] Singh S, Best C, Dunn S, Leyland N, Wolfman WL. (2013) Abnormal uterine bleeding in pre-menopausal women. J Obstet Gynaecol Can 35:473–5. Epub 2013/06/13. 10.1016/S1701-2163(15)30939-7 . 23756279

[15] Ministère des Affaires Sociales de la Santé et des droits des femmes. Guide méthodologique de production des informations relatives à l’activité médicale et à sa facturation en médecine, chirurgie, obstétrique et odontologie. 2015

[16] Bezin J, Duong M, Lassalle R, Droz C, Pariente A, Blin P, et al (2017) The national healthcare system claims databases in France, SNIIRAM and EGB: Powerful tools for pharmacoepidemiology. Pharmacoepidemiol Drug Saf 26:954–62. Epub 2017/05/26. 10.1002/pds.4233 . 28544284

[17] World Health Organization. International Classification of Diseases 10th Revision. Geneva: WHO, 2010.

[18] maladie Cndla. Open Medic: base complète sur les dépenses de médicaments interrégimes 2018 [cited 2018 18th December]. Available from: https://www.data.gouv.fr/en/datasets/open-medic-base-complete-sur-les-depenses-de-medicaments-interregimes/.

[19] Motta T, Lagana AS, Valenti G, VL LAR, Noventa M, Vitagliano A, et al (2017) [Differential diagnosis and management of abnormal uterine bleeding in adolescence]. Minerva Ginecol 69:618–30. Epub 2017/10/31. 10.23736/S0026-4784.17.04090-4 . 29082726

[20] Mullins TL, Miller RJ, Mullins ES. (2015) Evaluation and Management of Adolescents with Abnormal Uterine Bleeding. Pediatr Ann 44:e218–22. Epub 2015/10/03. 10.3928/00904481-20150910-09 . 26431240

[21] Fergusson RJ, Lethaby A, Shepperd S, Farquhar C. (2013) Endometrial resection and ablation versus hysterectomy for heavy menstrual bleeding. Cochrane Database Syst Rev:Cd000329. Epub 2013/11/30. 10.1002/14651858.CD000329.pub2 . 24288154

[22] Brandsborg B, Nikolajsen L. (2018) Chronic pain after hysterectomy. Curr Opin Anaesthesiol 31:268–73. Epub 2018/02/24. 10.1097/ACO.0000000000000586 . 29474214

[23] Clarke-Pearson DL, Geller EJ. (2013) Complications of hysterectomy. Obstet Gynecol 121:654–73. Epub 2013/05/03. 10.1097/AOG.0b013e3182841594 . 23635631

[24] Topsoee MF, Ibfelt EH, Settnes A. (2016) The Danish Hysterectomy and Hysteroscopy Database. Clin Epidemiol 8:515–20. Epub 2016/11/09. 10.2147/CLEP.S99465 . 27822093 PMC5094637

[25] Reid PC. (2007) Endometrial ablation in England—coming of age? An examination of hospital episode statistics 1989/1990 to 2004/2005. Eur J Obstet Gynecol Reprod Biol 135:191–4. Epub 2006/10/19. 10.1016/j.ejogrb.2006.08.008 . 17045729

[26] Amso NN, Fernandez H, Vilos G, Fortin C, McFaul P, Schaffer M, et al (2003) Uterine endometrial thermal balloon therapy for the treatment of menorrhagia: long-term multicentre follow-up study. Hum Reprod 18:1082–7. Epub 2003/05/02. 10.1093/humrep/deg206 . 12721188

