# Supplementary material for: The surgical treatment of idiopathic abnormal uterine bleeding: An analysis of 88 000 patients from the French exhaustive national hospital discharge database from 2009 to 2015
Source: PLoS One. 2019 Jun 11;14(6):e0217579. doi: 10.1371/journal.pone.0217579 (PMC6559634; doi:10.1371/journal.pone.0217579)
Supplement: S1 Table — (DOCX) [file pone.0217579.s001.docx]

The surgical treatment of idiopathic abnormal uterine bleeding:
an analysis of 88 000 patients from the French exhaustive national hospital discharge database from 2009 to 2015

SUPPLEMENTARY TABLES

S1 Table.

|  | 2G Surgery N = 7,863 | 1G Surgery N = 39,935 | Curettage N = 38,923 | Hysterectomy N = 23,163 | TOTAL N = 109,884 |
| --- | --- | --- | --- | --- | --- |
| Age (years) | 46 ± 4.5 | 46 ± 5.0 | 46 ± 5.4 | 46 ± 4.5 | 46 ± 5.0 |
| Hospitalisation  Overnight stay  Day hospitalisation | 1,321 (16.8%) 6,542 (83.2%) | 6,832 (17.1%) 33,103 (82.9%) | 8,141 (20.9%) 30,782 (79.1%) | 23,058 (99.5%) 105 (0.5%) | 39,352 (35.8%) 70,532 (64.2%) |
