# Supplementary material for: The surgical treatment of idiopathic abnormal uterine bleeding: An analysis of 88 000 patients from the French exhaustive national hospital discharge database from 2009 to 2015
Source: PLoS One. 2019 Jun 11;14(6):e0217579. doi: 10.1371/journal.pone.0217579 (PMC6559634; doi:10.1371/journal.pone.0217579)
Supplement: S2 Table — (DOCX) [file pone.0217579.s002.docx]

The surgical treatment of idiopathic abnormal uterine bleeding:
an analysis of 88 000 patients from the French exhaustive national hospital discharge database from 2009 to 2015

SUPPLEMENTARY TABLES

S2 Table.

| CCAM code | French title |
| --- | --- |
| 2G (2^nd^ generation endometrial ablation techniques such as radiofrequency, laser…) | |
| JKND001 | Destruction de la muqueuse utérine par thermocontact, par voie vaginale |
| 1G (1^st^ generation endometrial ablation techniques, such as loop resection) | |
| JKNE001 | Abrasion de la muqueuse de l'utérus [Endométrectomie], par hystéroscopie |
| Curettage | |
| JKGD002 | Curetage de la cavité de l'utérus à visée thérapeutique |
| JKQE001 | Hystéroscopie avec curetage de la cavité de l'utérus (without JKND001) |
| JKGD003 | Curetage de la cavité de l'utérus à visée diagnostique (with anesthesia) |
| Hysterectomy | |
| JKFC002 | Hystérectomie subtotale, par cœlioscopie |
| JKFC006 | Hystérectomie subtotale avec annexectomie unilatérale ou bilatérale, par cœlioscopie |
| JKFC005 | Hystérectomie totale, par cœlioscopie |
| JKFA018 | Hystérectomie totale, par cœlioscopie et par abord vaginal |
| JKFA026 | Hystérectomie totale, par abord vaginal |
| JKFC003 | Hystérectomie totale avec annexectomie unilatérale ou bilatérale, par cœlioscopie |
| JKFA006 | Hystérectomie totale avec annexectomie unilatérale ou bilatérale, par cœlioscopie et par abord vaginal |
| JKFA005 | Hystérectomie totale avec annexectomie unilatérale ou bilatérale, par abord vaginal |
| JKFA024 | Hystérectomie subtotale, par laparotomie |
| JKFA032 | Hystérectomie subtotale avec annexectomie unilatérale ou bilatérale, par laparotomie |
| JKFA015 | Hystérectomie totale, par laparotomie |
| JKFA028 | Hystérectomie totale avec annexectomie unilatérale ou bilatérale, par laparotomie |
