# Supplementary material for: The surgical treatment of idiopathic abnormal uterine bleeding: An analysis of 88 000 patients from the French exhaustive national hospital discharge database from 2009 to 2015
Source: PLoS One. 2019 Jun 11;14(6):e0217579. doi: 10.1371/journal.pone.0217579 (PMC6559634; doi:10.1371/journal.pone.0217579)
Supplement: S3 Table — (DOCX) [file pone.0217579.s003.docx]

The surgical treatment of idiopathic abnormal uterine bleeding:
an analysis of 88 000 patients from the French exhaustive national hospital discharge database from 2009 to 2015

SUPPLEMENTARY TABLES

S3 Table.

| ICD-10 code | Wording |
| --- | --- |
| N800 | Endometriosis of uterus |
| N850 | Endometrial hyperplasia |
| N920 | Excessive and frequent menstruation with regular cycle |
| N921 | Excessive and frequent menstruation with irregular cycle |
| N924 | Excessive bleeding in the premenopausal period |
| N925 | Other specified irregular menstruation |
| N926 | Irregular menstruation, unspecified |
| N938 | Other specified abnormal uterine and vaginal bleeding |
| N939 | Abnormal uterine and vaginal bleeding, unspecified |
